# Airbag Vests in Equestrian Sports: Is Use Associated with Harm?

**DOI:** 10.1007/s10439-024-03507-y

**Published:** 2024-07-02

**Authors:** Catherine Meyer, Fernanda Gabriel, Kevin Schrum, Michele Hollis, Margo Short, Sara Gould

**Affiliations:** 1 University of Alabama at Birmingham School of Medicine, Birmingham, AL USA; 2 Department of Biomedical Engineering, University of Alabama at Birmingham, Birmingham, AL USA; 3 Department of Mechanical and Materials Engineering, School of Engineering, University of Alabama at Birmingham, Birmingham, AL USA; 4 CMO HollisMed LLC, Wellington, FL USA; 5 University of Kansas Medical Center, Kansas City, KS USA; 6 Birmingham Veterans Administration Health Care Service (VAHCS), Birmingham, AL USA; 7 Division of Sports Medicine, Department of Orthopedics, University of Alabama at Birmingham, Birmingham, AL USA

**Keywords:** Equestrian sport, Airbag vest, Protective clothing, Athletic injuries, Body armor

## Abstract

Airbag vests (AV) are increasingly popular in equestrian sports. The efficacy of AV in protecting against serious injury has not been adequately analyzed, nor have product testing standards been established. This study provides an overview of current research to understand AV efficacy and future areas of improvement. A systematic review applying the PRISMA framework, NIH Study Quality Assessment, and CEBM Level of Evidence was conducted. Employing variations of “equestrian sport,” “powered two-wheeled vehicle,” “thoracic injury,” “chest deflection,” “airbag vest,” and “safety vest,” 18 articles were identified for data collection from three recognized research databases and citation searching. In laboratory settings, the ability of AV to protect against thoracic injuries was variable based on concurrent foam-based safety vest (SV) usage, impact speed, and impact mechanism. Studies that examined equestrian falls with AV found an association with increased injury rates and risk. SVs were shown to provide inconclusive efficacy in protecting against injuries in experimental and cohort studies. Protective capabilities depend on material, temperature, and impact mechanism. Further limiting use, equestrians reported not wearing, or incorrectly wearing SV due to unknown benefits, low comfort, and ill fit. In equestrian sports, based on published literature to date, AV have not been associated with a reduction in injury. AV appear to be associated with an increase in the risk of serious or fatal injuries in certain settings. However, research in this area is limited and future, large-scale studies should be conducted to further evaluate the efficacy of the air vests.

## Introduction

Equestrian is the umbrella term for sports involving riding on horseback which range from Olympic modalities (show jumping, dressage, and eventing) to horse racing. Horses weigh an average of 1500 lb., can travel up to speeds of 40 mph, and a single kick may deliver 1000 N of force [1]; this, added to their unpredictable behavior, may increase the risks carried from dealing with horses. Statistics show that one in every five riders sustains some form of injury per year and, surprisingly, the rate of serious injury per number of riding hours is estimated to be higher for horseback riders (1 in 350 h) than for motorcyclists (1 in 7000 h), and automobile racers (1 in 833 h) [2]. The most common injuries sustained in equestrian sport are to the head/neck and thoracic regions, mainly due to a fall from the horse [3–5].

Therefore, much attention has been focused on the well-being of riders in equestrian sports, including national and international safety regulations, advances in protective devices such as helmets and safe-release stirrups, the mandatory use of safety vests (SVs) in the cross-country phase of eventing in 1996 [6], the helmet mandate introduced for almost all equestrian disciplines in 2021 [7], and the development of new technologies such as airbag vests (AV). AV technology has been used by motorcyclists since late 1990s [8], but has recently been adopted by equestrians, even though the efficacy against severe thoracic injuries resulting from falling from the horse remains controversial. An AV is worn on top of riding clothes, and it is attached to the saddle by a lanyard after getting on the horse. It is used with a removable CO_2_ air cartridge that allows the whole vest to inflate in 0.2 s after an event of a forceful separation between the lanyard connecting the horse and rider [9].

AV are a subcategory of SV. SVs are foam-based body protectors made from some combination of dense foam, tactical materials, or ballistic nylon [9] and are intended to protect the rider’s vital organs in an event of a fall. SVs have specific testing regulations and are governed by 3 international safety standards: CE EN13158, ASTM F1937, and ASTM F2681. The European Equestrian Standard EN13158 2018 [10] specifies the requirements and test methods for the coverage, sizing, adaptability and adjustability, restraint, ergonomics, construction, innocuousness, and performance to be provided by protective jackets, body, and shoulder protectors. The impact tests are designed to represent the hazards of falling from a horse, of being hit by a hoof or even by landing over a narrow pole.

Anvils and guard ring system are designed to represent body parts profiles and some of their responses to impact; this way, each anvil is mounted onto a stiff load cell or force transducer and a flat face impactor is used to hit them. The safety vests to be tested should be laid flat on the guard ring and individual peak force values should be recorded; the mean peak force for all impacts on the same vest should be calculated. The testing parameters are defined according to the level of performance of the rider and the drop height of the impactors above the protective clothing being tested should be adjusted so that the impact velocity provides an impact energy. See Table 1.

**Table 1:** Impact energy for testing performance levels 1, 2 and 3 [10]

Test conditions	Impact energy for the performance level (J)		
	Level 1 Lowest level of performance for garments worn by licensed jockeys only	Level 2 Category of garments for low risk	Level 3 Category of garments for high risk
Flat impactor on body protectors and the torso region of protective jackets. Guard ring, 0 mm.	25	30	35
Narrow bar impactor on body protectors and the torso region of protective jackets. Guard ring, 10 mm.	20	32.5	45
Wide bar impactor on shoulder protectors and the shoulder region of protective jackets. No guard ring.	60	60	60
With accuracy of ± 5%			

In order to be in accordance with the standards defined by the impact tests, the mean peak force recorded below the anvil in each test for body protectors of all types and all of their points added together should be < 4 kN, and any single value should be ≤ 6 kN [10]. The biofidelity of anvil-type testing for other types of equestrian safety equipment has been called into question; however, testing standards for equestrian safety equipment still rely on this methodology [11]. Over the last decade, various attempts have been made to modify existing SV standards to govern AV. SATRA M38: Issue 3 February 2015 air vest standard was designed as a research project by UK-based SATRA Laboratories in collaboration with the British Racehorse Authority; however, SATRA is no longer testing AV. Currently, there is a European committee working to develop a new AV testing standard, which will update the prior European Equestrian SV standard EN13158 2018 to incorporate NF S72-800:2022 [12], which was developed by Aliénor, a French Laboratory. To date, there are no published testing standards for AV.

Given the lack of independent research on AV, the absence of internationally accepted testing standards, and the wide-spread adoption of this technology, there is a gap between research and implementation. This study aims to analyze the efficacy as well as rider use and opinions regarding safety vests and airbag vests in equestrian sports, utilizing quasi-experimental studies on powered two-wheeled vehicle accidents as supplement.

## Materials and Methods

PubMed, Embase, and SCOPAS platforms were queried with no limits on article type, language, or date. The search terms “equestrian sport,” “powered two-wheeled vehicle,” combined with the terms “trunk injury,” “safety vest,” and “airbag vest” were utilized to extract data. The full search strategy is included as an attachment. The search strategy and inclusion/exclusion criteria were expanded outside of strictly equestrian sports/injuries due to the limited data available in the equestrian realm.

Inclusion criteria included any article with the correct activity, injury, and protective equipment. Additionally, articles concerning jockey or rough stock rodeo rider injuries were reviewed and included even if use of protective equipment was not specifically defined, as SVs are required in all these competitions. Studies were excluded that examined activities, injuries, and protective equipment that were not listed in the inclusion criteria; as well as studies that examined injuries involving only the horse. Experimental studies on powered two-wheel vehicles that examined chest/sternum deflection or body acceleration were also included; all other formats of studies on this subject were excluded.

Search results were exported to Covidence, and any duplicated articles were removed. A PRISMA flowchart was created and maintained. Each study title and abstract were screened by two authors, with a third author settling any discrepancies. Full texts of accepted studies were screened in the same manner. Of the 18 studies that were reviewed through this process, 5 specifically addressed airbag vests. Three Motocross studies were also analyzed, including one observational study and two laboratory-based studies. NIH Study Quality Assessment and CEBM Level of Evidence tools were utilized by reviewers during full-text review [13, 14].

Additional articles were found and included through examining accepted article’s reference lists and hand searches. Additional articles were exported into Endnote 21 and added to Covidence where they underwent the screening protocol described above. Any non-English articles were translated into English using the Google Translate Chrome add-on, which has been described as an accepted practice for literature reviews [15, 16].

## Results

**Fig. 1:**
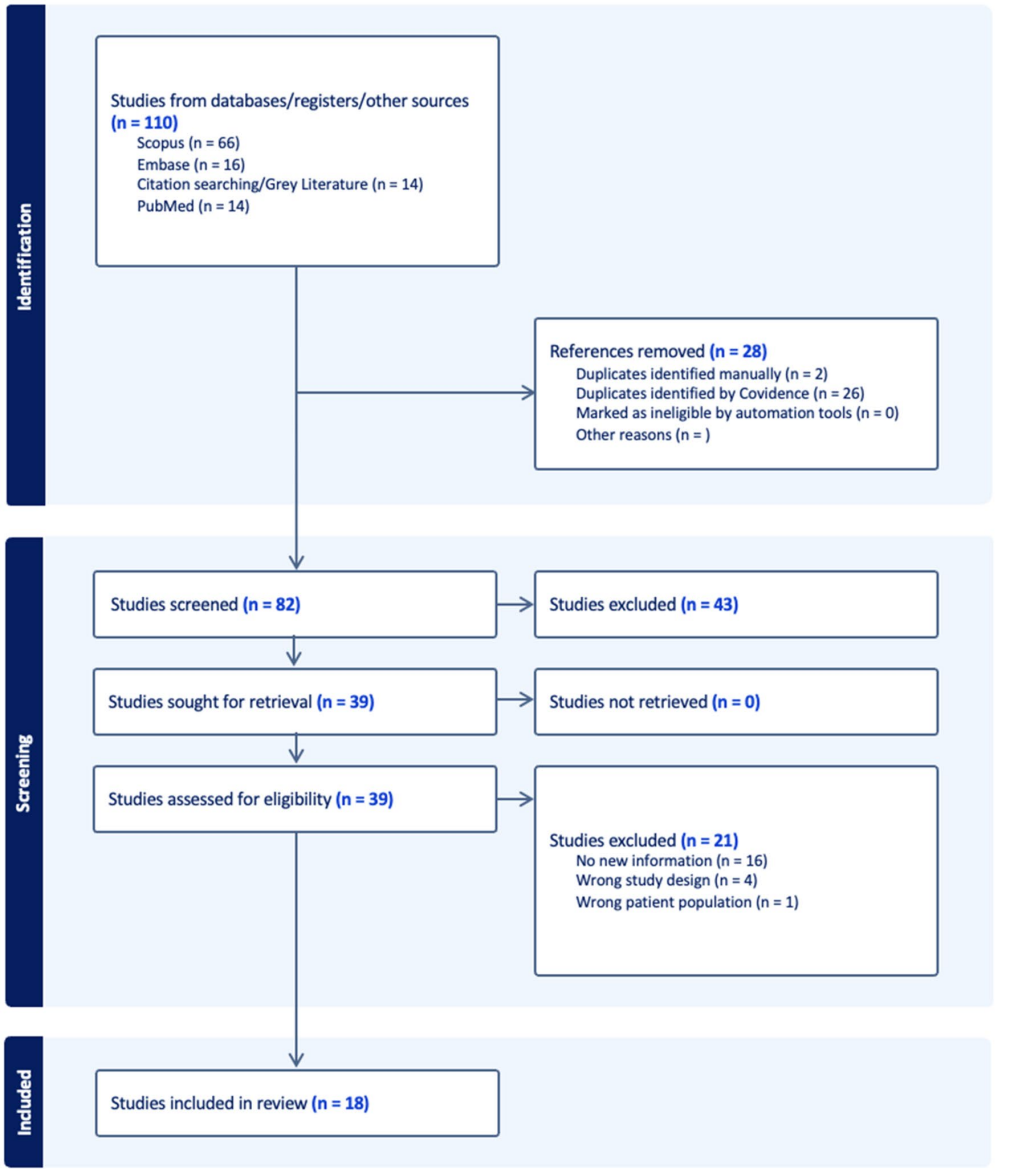
PRISMA flowchart summarizing systematic review results produced by Covidence [34]

We found 110 studies from database, citation, and gray literature searching. Following removal of duplicates, 82 studies were screened resulting in 43 being excluded. During full-text assessment of 39 studies, 18 were identified for inclusion in the present review [6, 17–33]. Whether or not the vests included in the 18 studies found met testing requirements is largely unknown as the brand and protection levels were not reported in the observational studies and results were variable in the experimental studies. The PRISMA flowchart produced by the Covidence systemic review software is demonstrated in Fig. 1.

### Airbag Vests

Laboratory studies reported overall protective effects of AV, though the reproducibility of these studies beyond the laboratory setting is unknown. One equestrian laboratory-based study reported that AV were able to absorb higher forces than SV [17]. This study used an 8 cm (3.14 inch) 2.5 kg drop weight to test impact onto a piezoelectric force sensor beneath the equestrian vests. The AV were manually inflated but only to 35 kPa (5.1 psi), rather than the manufacturer’s designed pressure, which was unknown. AV were also tested in combination with SV in this study. The authors reported the mean impact force for all SV/AV combinations was significantly lower than those derived from SV alone; with one brand of SV demonstrating an improvement from 79 to 94% reduction of impact force when combined with an AV [17]. The authors measured acoustic shock associated with inflation and no potential for ear injury was identified [17].

In a novel approach, another laboratory-based study dropped a horse cadaver onto a mannequin and measured peak chest deflection [18]. The authors reported that the combination of an AV and SV reduced peak chest deflection from 77 to 66 mm, compared to a SV alone [18]. This corresponds to a severe chest injury reduction risk from 94 to 81%, per their report [18].

In contrast, the observational studies did not demonstrate injury reduction associated with AV use [19–21]. On the contrary, these observational studies reported that AV use was associated with an increase in both the risk and the rate of injury among equestrians.

In a study of International Federation for Equestrian Sports (FEI) level competitions, equestrians wearing an AV had a 1.7 times increased risk of sustaining severe or fatal injury over riders not wearing an AV [20]. Another study on cross-country competitors in France analyzed injury rates resulting from falls during competitions [21]. The authors reported a 13.7% injury rate for equestrians who fell while using AV, compared to 9% for riders who fell while not wearing an AV [21]. Finally, a video analysis of equestrian falls sustained during British Eventing and International Equestrian Federation competitions reported an increased number of high-risk landings in equestrians using AV (*p* = 0.007; OR 6.5) [19]. Late inflation of the airbag vests occurred with 25% of recorded falls among equestrians utilizing AV [19]. The phenomenon of late inflation was noted to occur more frequently with simultaneous horse and rider falls, as opposed to falls of the rider alone [19].

The level of evidence (LOE) and quality of evidence (QOE) of the five AV studies discussed are presented in Table 2.

**Table 2:** Airbag vest publication scoring

Author	Publication year	Publication type	LOE	QOE
Chiodetti [21]	2015	Prospective cohort study	2b	Fair
Nylund [20]	2019	Case control	3b	Fair
Nylund [19]	2021	Cross-sectional	2c	Fair
Ade [17]	2016	Experimental/case series	4	Fair
Hynd [18]	2016	Experimental/case series	4	Poor

### Safety Vests

No clear consensus on SV efficacy of injury reduction was noted in our analysis of 10 manuscripts on the subject, with some studies reporting decreased rates of injury and others reporting no effect [6, 22–29]. In a survey study of 363 German show jumpers, researchers found that riders who always or often wore SV experienced less spinal injuries than those who never or occasionally wore SV (*p* = 0.017) [22]. On the other hand, a case-control study of 92 German equestrians found that use of a SV was not associated with a lower risk of suffering an injury to the torso (OR 1.18, 95% CI (0.50, 2.81), *p* = 0.707)[23]. There were no SV studies that reported an increased risk of injury with this type of vest. The studies reported that protective capabilities were contingent on the vest composition, the environment it was used in, and the testing mechanisms [25, 28, 29]. Equestrians cite high costs, unknown benefits, and discomfort as deterrents for utilizing SV [6, 30].

### Airbag Vest in Powered Two-Wheel Vehicles

Research on AV in the field of motorsports has suggested that in this domain, under certain conditions, AV offer protection against injury. One study reported that use of SV with or without AV decreased odds of sustaining a back fracture or spinal cord injury compared to those not wearing any form of body protection [31]. In two experimental studies, the protective capacity of AV against serious thoracic injuries declines with accidents at speeds over 40 km/h [32, 33]. The authors suggest that the vest capabilities are contingent upon their material features and the fall/testing mechanisms and parameters [32, 33].

## Discussion

In experimental studies, AV appear promising in their ability to protect riders from sustaining serious or fatal trunk injuries. In equestrian practice, however, AV appear to be associated with an increase in the risk of serious or fatal injuries.

The discrepancy maybe be explained by limitations in the laboratory setting. For example, one study reported that over half of their AV data was lost during analysis, so the results only represented a fraction of the total data that had initially been collected on AV [18]. Another study utilized anvil and drop testing of a weight; however, neither the anvil nor the dropped weight approximates the physical properties of the surfaces equestrians commonly contact during impact [17]. Ballistic studies on various sport ball impacts to the human thorax have reported that the physical properties of the projectile have vast influence on the forces deployed at impact [35]. Therefore, studies which rely on projectile-type drop tests may not accurately replicate the forces seen in equestrian falls.

The standards to which AV have been tested may also prevent translation to equestrian practice. The current EN 13158 2018 standards were designed to test SV and have been adapted by various laboratories and industries to evaluate AV [17, 36]. However, at the time of writing, no information was available on assessments for timing of airbag deployment, performance of airbags at extremes of ambient temperatures, or effect of the AV lanyard attachment to the saddle on fall trajectory. The pressure of the gas used to inflate the AV is directly proportional to temperature to a higher degree than solid materials, so special consideration of these properties should be assessed.

Literature suggests that while AV may reduce the peak chest deflection and mean impact force experienced by the user, they do not do so to such an extent that the user would be protected from sustaining a serious or fatal trunk injury following a high force impact. In fact, multiple professional societies no longer recommend protective equipment for patients with solitary kidney, due to the lack of evidence supporting any protective effects against solid organ injury during sport [37, 38]. Additionally, the AV may introduce variables which actually increase injury risk, such as late airbag inflation during a combined horse and ride fall, as reported by Nylund et al. [19], decreased ability to perform tuck and roll maneuvers upon impact to roll away from the horse, or injury from landing on the hard canister that contains the gas cartridges within the vest.

AV inflate along the torso, decreasing natural curvature of the spine, which may increase injury risk upon impact. Professional jockeys have noted that bulky SV hinder their ability to perform tuck and roll maneuvers on impact [25]. Some authors have also suggested that the lanyard attaching the equestrian to the saddle may actually prevent normal fall trajectory and increase risk of trampling injury by the rider’s own horse [20]. These authors reported that pull forces ranging from 150 and 593 N had to be affected to trigger AV inflation and hypothesized that this would be sufficient force to alter the kinetics of an equestrian’s fall [20]. Further, AV are typically tested on adult-sized models, so individuals with smaller masses (youth) may not trigger AV detachment/inflation in the same manner, which could lead to late deployment and increased injury risk.

Downstream mechanical implications of AV inflation need to be considered, as well. Jockeys have reported that SV can come into contact with their helmets during certain maneuvers, leading to visual impairment [30, 39]. They have voiced concern over this interaction and its potential impact on helmet function [30]. Additionally, medical professionals covering races report that the vests can be difficult to remove in the event of injury, leading to delayed care [39]. Since some brands of AV attempt to provide cervical spine protection by inflating around the neck, consideration of how the AV will interact with the helmet is imperative. Certain helmets, such as those employing multi-directional impact protection systems (MIPS) depend on the ability to pivot slightly upon impact to diminish rotational forces transmitted to the brain. Combining an AV that seeks to stabilize motion, with a helmet that relies on some degree of motion for function may result on deleterious consequences in function.

AV protective capabilities were documented in powered two-wheeler vehicle studies; however, no study greatly supports translation of this effect to equestrian sports. In Serre’s study, it was found that below 40 km/h, airbag vests appeared to be efficacious in preventing trunk injuries of all levels [32]. Similarly, Thollon found that while pendulum load reduction was inversely related to impact speed, trunk injury risk was only observed at an impact speed of 11.11 m/s (40 km/h) [33]. Further equestrian studies, however, are required to see if these findings are translatable for application in equestrian sport. When a motorcyclist falls from the motorcycle, the fall height is under 3 ft. A fall from the back of a horse is substantially higher. Coupled with higher speeds, the fall from a horse could be a much more severe loading case for the AV. Speed is an important component in many equestrian competitions which require a horse to gallop (44 km/h average). Equestrian competitions which do not rely on speed may see a benefit in AV use, notwithstanding consideration of the other physical and mechanical implications discussed above.

AV have not been shown to decrease injury risk in equestrian sports. Use of AV has been associated with increased risk of injury in some equestrian competitions. Improving injury surveillance through large-scale monitoring programs that are customized to the nuances of equestrian sports would address the urgent need to further characterize AV function and safety profiles.
